# Interdisciplinary Fascia Therapy: A Proof-of-Concept Pilot Study for a New Myofascial Approach for Chronic Low Back Pain

**DOI:** 10.3390/jcm13237226

**Published:** 2024-11-28

**Authors:** Christopher M. Gordon, Victoria Dugan, Christina Hörmann, Pedro Montoya

**Affiliations:** 1 CIT Research Institute, 70597 Stuttgart, Germany; 2 Fascia Research Group, Division of Neurophysiology, University of Ulm, 89081 Ulm, Germany; 3 Research Institute of Health Sciences (IUNICS), University of the Balearic Islands, 07122 Palma de Mallorca, Spain; pedro.montoya@uib.es

**Keywords:** interdisciplinary fascia therapy, chronic low back pain, myofascial trigger point release, heart rate variability, brief pain inventory, paced breathing therapy

## Abstract

**Background/Objectives:** Chronic low back pain (CLBP) is highly prevalent and relevant in all medical fields. This study evaluated the safety and effectiveness of interdisciplinary fascia therapy (IFT) for CLBP, focusing on its potential to reduce pain intensity, disability, and regulate autonomic nervous system (ANS) activity. **Methods:** Nine participants with CLBP each underwent nine sessions of IFT, twice weekly. The intervention involved a 12-grip manual sequence targeting deep paravertebral myofascial structures, complemented by heart rate variability (HRV) biofeedback training twice daily for 15 min. Pain and quality-of-life measures were assessed using the Brief Pain Inventory (BPI) questionnaire at baseline, mid-treatment (4th session), and post-treatment (9th session). HRV metrics were monitored with a 24 h ECG Holter device before and after the treatment period. Statistical analyses included paired *t*-tests, Wilcoxon signed-rank tests, and Cohen’s d for effect size. **Results:** Significant reductions in pain levels were observed across all subjective BPI measures, including momentary, strongest, minimal, and average pain scores (*p* < 0.001), with 83% and 87% reductions in pain intensity and disability, respectively. Quality-of-life indicators such as mood, sleep, and enjoyment of life showed significant improvements (*p* < 0.001). While only one HRV metric (rMSSD) achieved statistical significance, other HRV measures indicated medium to large effect sizes, suggesting favorable trends in ANS regulation. **Conclusions:** IFT demonstrated significant effects on subjective BPI pain reduction and quality of life, alongside potential regulatory impacts on ANS activity in individuals with CLBP. These results support the use of IFT as an effective intervention for pain management in CLBP and ANS regulation, meriting further exploration.

## 1. Introduction

Chronic low back pain (CLBP) is a widespread and largely underestimated health issue that negatively impacts the well-being and functional capabilities of individuals as well as their contributions to society. More than 70% of people experience back pain at some stage in their lives, and 5–15% endure the condition chronically and for the long-term [1]. Pain is characterized as chronic if it lasts 3 months or more in duration [2]. Lumbar back pain has an impact on society as healthcare costs increase and productivity decreases. Capacity for work is markedly diminished when both the physical and psychological capabilities of the person are compromised by being exposed to prolonged pain [3]. Hall et al. [4] cite chronic back pain as one of the top seven prompts for general practitioner consultations in North America, whilst Srbely et al. [5] found that it was the second most common in Canada.

Medication-based approaches have long received the greatest attention for the treatment of CLBP.

A range of surgical interventions, including spinal column manipulations, have also been used in CLBP therapy [6,7,8]. In addition to the high costs that are incurred, surgical interventions carry the risk of infection and other side effects that delay recovery, lengthen the patient’s absence from productive work, and compromise their enjoyment of life.

CLBP is also positively correlated with various forms of disk disease and degeneration [9]. Nevertheless, as many as 85% of patients had asymptomatic MRI findings that confirmed the presence of disk degeneration, but without pain [10,11,12]. Recent evidence suggests myofascial trigger points (MTP) may be present in as many as 95% of people with chronic pain disorders [13]. MTPs are described as irritable lumps in a narrow band of skeletal muscle and causing pain when muscles contract, stretch, or are otherwise stimulated, which may lead to pain at a referred distance. MTPs are distinguished as active or latent. Active MTPs are those where local and referred pain symptoms are reported by patients. Latent MTPs have the same clinical findings as active MTPs, but do not cause pain [14]. Biochemical findings support the clinical differentiation between active and latent MTPs, as higher concentrations of chemical mediators such as bradykinin, substance P, and/or serotonin have been found in active MTPs rather than in latent MTPs and non-MTPs variants [15].

The pivotal role of the myofascial system in pain disorders, unfortunately, has not received the serious attention it merits, with it being excluded in differential diagnostic processes for the treatment of CLBP.

Myofascial trigger point therapy, a primary component of myofascial release, is another treatment methodology for CLBP. By releasing tension in the trigger points and increasing blood flow to it and the surrounding tissue, the trigger point becomes a less likely cause of pain for that area and referred pain. Only a few studies have investigated the effectiveness of osteopathic treatment for CLBP [16,17,18,19]. Other treatment options, such as biofeedback training, show a highly significant and positive change in pain levels; as does acupuncture in the treatment of back pain, which has gained attention from scholars [20,21,22].

CLBP generates not only physiological symptoms but psychological ones as well, such as anxiety and depression [23,24,25]. Recent studies of ANS disorders show an involvement of ANS dysregulation in the symptoms of chronic muscle pain. These studies have also shown the effectiveness of heart rate variability (HRV) training for increasing HRV and reducing the symptoms of various conditions characterized by ANS aberration [26]. HRV is the term for the change in heartbeat frequency [27] and is often utilized as a “stress index” [28] because HRV analysis can provide insight into ANS functioning. HRV training seems to stimulate the vagus nerve, the main nerve of the parasympathetic nervous system of the ANS, leading to a reduced production of pro-inflammatory substances responsible for generalized systemic inflammation [29,30], which can result in chronic pain [31]. Heart rate changes in accordance with respiration; for example, during exhalation, blood pressure increases and the heart rate decreases [32]. This physiological phenomenon is known as Respiratory Sinus Arrhythmia (RSA) [33] and is why HRV can be trained through breath work. HRV observation can be used to assess cardiac vagus tone and represents cardiac regulation, associated with the ability to self-regulate at cognitive, emotional, social, and health levels [34].

Improving quality of life is additionally a positive psychology tool for increasing emotional resilience in chronic pain management, fostering mindfulness, and being intentional about ones physical and mental health can shift a participant’s focus away from pain intensity and disability and decreases pain-related stress. This is an additional aspect of this study’s comprehensive approach to pain management and relief [35].

The RSA is governed by the depth of breaths taken and the respiratory rate of a patient. The resonance frequency of the baroreceptors in humans is in a range of approximately 0.1 Hz [36], which in most cases corresponds to a respiratory rate of six breaths per minute. Paced breathing training (PBT) thus can be used to modulate respiratory rate with the assistance of a specially adapted acoustic pacemaker [37]. A reduction in stress occurs with the increased activity of the parasympathetic branch of the ANS [38,39,40,41]. Enhancing the functioning of the ANS is the goal of HRV training because it encourages the patient to develop a more relaxed focus and to handle stress more effectively. Previous work has revealed that a breathing pattern of 5.5 breaths per minute optimally improves HRV, while simultaneously stimulating vagal nerve activity [42].

HRV training has demonstrated efficacy across various psychological dimensions. However, its potential physiological impact, particularly when integrated with myofascial therapy for chronic pain conditions, presents a novel area of study [43]. This pilot study was designed to provide a preliminary evaluation of the efficacy of the interdisciplinary fascia therapy (IFT method), combining the myofascial trigger point release (MTPR) method and paced breathing HRV training, to manage chronic pain in nine participants over a five week period.

## 2. Materials and Methods

### 2.1. Participants

**Figure 1:**
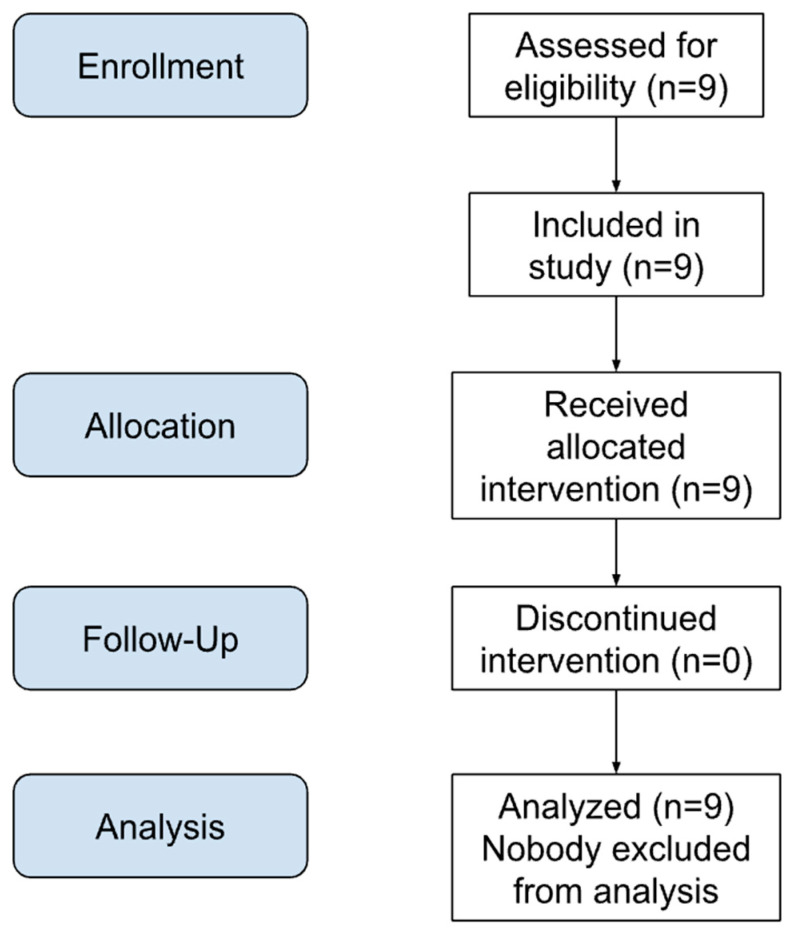
Consort flow diagram.

Potential eligible patients were identified by an orthopedic surgeon from those attending his clinic and experiencing chronic lumbar back pain with additional severe pain in the entire lumbar region. These patients were interviewed by telephone to affirm they met predetermined inclusion and exclusion criteria. Essential information about the purpose and the nature of the study was sent to each respondent prior to study enrollment. Due to absent reliable data regarding the effects of MTPR, with or without the simultaneous application of HRV with CLBP, we were unable to estimate an appropriate sample size and arbitrarily targeted the first 9 consecutive patients meeting our criteria for this preliminary investigation (Figure 1).

#### 2.1.1. Inclusion Criteria

Inclusion criteria were as follows

Adults aged 18 or above.

Chronic low back pain experienced at least once a week for the past three months.

A minimal pain rating of 3 in the last 24 h on a 0–10-point scale, where 0 = no pain and 10 = maximum imaginable pain, which was obtained from a single question in the Brief Pain Inventory (described below).

#### 2.1.2. Exclusion Criteria

Acute pain, such as that caused by intervertebral disk hernias, local, or general inflammation, was the primary exclusion criterium. Other exclusion criteria were as follows: neurological disorders and systemic conditions; conditions affecting lower extremity function; individuals currently undergoing chemotherapy and/or radiosurgery or intending to do so within 12 months; presence of a major mental health condition for which they were receiving psychotherapeutic or medical interventions; suicidal ideation or intentions; drug abuse and/or addictions; and being pregnant or likely to become pregnant during the course of the study. Participants with lower extremity prosthesis, rheumatic complaints, or chronic pulmonary or cardiovascular disease, as well as those for whom a 45 min physical therapy session was contra-indicated in the opinion of an internist, were also excluded from the study. These exclusion criteria were chosen to ensure participant safety and the study’s focus on chronic low back pain (CLBP) without confounding factors, including preventing influences on pain perception and autonomic regulation.

All nine screened volunteering participants successfully met the above initial criteria and were subject to a second round of screening that included an examination by an orthopedist and a physiotherapist. All participants were advised that all data collected during screening or treatment would be anonymized in accordance with the Federal Data Protection Act. Prior to signing a consent-to-participate form, they were informed that this study does not have ethics review board approval and reminded of their right to withdraw voluntarily from the study at any time without any penalty. This study was conducted in a manner consistent with the Declaration of Helsinki.

Throughout this study, all participants were required to cease any and all alternative pain management or therapeutic interventions, for instance other therapeutic work or medications.

### 2.2. Intervention

#### IFT Method

**Figure A1:**
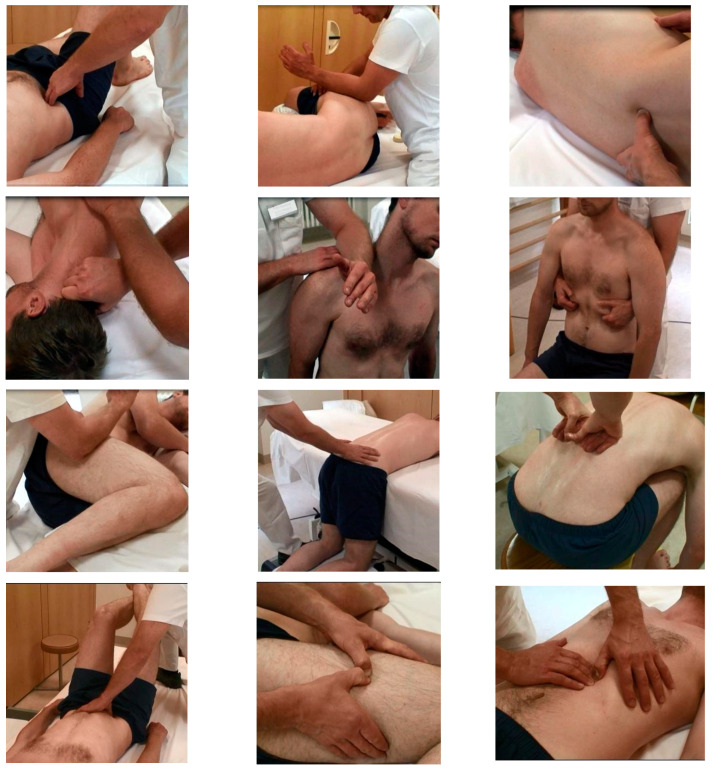
Grip, standardized, manual sequence (nine treatments of 60 min, twice per week).

Prior to the start of this study, all treating therapists received an eight-day training course in the IFT method from the developer of the method (C-MG), where they learned the protocol. They continued to receive close supervision throughout the duration of the study. The IFT method is composed of manual therapy and HRV training as described below. Each of the nine subjects was treated by one of the three trained physiotherapists during a total of nine 60 min sessions, spaced twice a week, over five weeks. The interventions included a specific standardized 12-grip sequence (Appendix A Figure A1). The 12-grip sequence is designed to sequentially release tension across specific myofascial structures from the lumbar to cervical regions, aiding in pain relief and ANS regulation. The sequences started with the myofascial release method of trigger points of the following muscles: gluteal; tensor fasciae latae; and the erector trunci, rototores, and multifidi.

**Figure 2:**
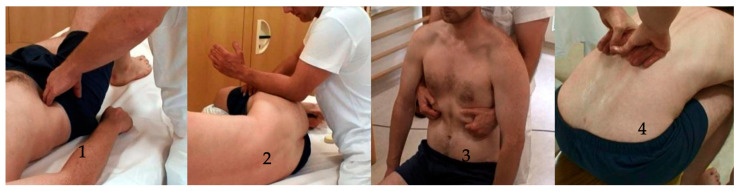
Excerpt of the standardized 12-grip protocol.

This first part of the intervention was followed by the release of the myofascial structures and trigger points in the scaleni and sternocleido-mastoidius. All muscles from cranium to sacrum were treated in the myofascial roll-down technique for the spine (see Figure 2, picture 4), which requires the patient to be in a sitting position and to bend forward slowly. The entire session was completed by an MFR (Myofascial Release) oscillation to the occiput with a rhythmic oscillation of the occipital bone.

### 2.3. Paced Breathing Training/HRV

Participants were additionally asked to complete a 15 min HRV training session twice a day at home during the five-week intervention. Participants were given CDs containing music designed to oscillate at a specific rhythm, guiding them to maintain a breathing rate of 5.5 cycles per minute, with equal durations for inhalation and exhalation [42]. CDs with music-based breathing exercises, designed to enhance HRV by encouraging paced breathing, were provided, along with written instructions, to augment treatment effects. Participant adherence was monitored through regular check-ins with each subject, in addition to their agreement to send photos of each treatment when it occurred.

### 2.4. Outcome and Process Measures

#### 2.4.1. Outcome: Brief Pain Inventory (BPI)

The German version of the Brief Pain Inventory (BPI) questionnaire [44], originally created by Charles S Cleeland in 1991, was used to determine levels of subjective pain intensity and disability (i.e., restrictions impacting everyday life and activities). This translated version of the BPI corresponds well with the original version and has demonstrated good validity and reliability. Four items on The BPI ask patients to rate the intensity of their pain, on scales ranging from 0 (“no pain”) to 10 (“pain as bad as you can imagine”): “worst” in the last 24 h, “least” in the last 24 h, “average” overall (no time period specified), and “right now”. An overall pain intensity score is obtained by averaging the values of these 4 individual ratings. Measures for pain interference over the past 24 h were obtained for the following seven daily activities: general activity, mood, walking ability, normal work, relations with other people, sleep, and enjoyment of life. Each item is rated on a scale from 0 (“does not interfere”) to 10 (“completely interferes”). An overall pain interference score may be obtained by averaging the individual ratings for each item. The BPI was administered before and after treatment concluded.

#### 2.4.2. Process: Heart Rate Variability (HRV)

A 24 h heart rate variability (HRV) measurement was obtained with an EKG Holter HRV measurement device (Autonom Health, Klosterneuburg, Austria) both before and after treatment completion. The following parameters were extracted from this HRV measurement:

#### 2.4.3. Standard Deviation of Beat-to-Beat or NN Intervals (SDNN) [ms]

The SDNN is a measure of changes in heart rate over long periods. The higher this value, the greater HRV and, therefore, the better assumed cardiovascular health and adaptability.

#### 2.4.4. Proportion of Successive Beat-to-Beat or NN Intervals That Differ by More Than 50 ms (pNN50) [%]

This assesses the percentage of consecutive RR intervals that differ by more than 50 ms. This value has a positive correlation with vagal tone; as this value increases, it can be assumed that the vagal tone is more active.

#### 2.4.5. Root Mean Square of Successive Differences (RMSSD) [ms]

The RMSSD expresses how much the heart rate changes from one heartbeat to the next. This value also has a positive correlation with vagal tone; as this value increases, it can be assumed that the vagal tone is more active.

#### 2.4.6. Frequency Domain Parameters

High-frequency (HF) power (ms^2^) is the power density spectrum in the frequency range from 0.15 to 0.40 Hz and provides a measure of vagal tone. Low-frequency (LF) power is the power density spectrum in the frequency range from 0.04 to 0.15 Hz and is also an indicator for vagal activity/baroreflex activity. Although various frequency domain parameters can be usually obtained, the device for the present study only provides these two measures of power (HF and LF).

#### 2.4.7. Biological HRV Age [Years]

HRV is an age-dependent variable, with values tending to decrease with age. To determine the biological HRV age, the age at which exactly 50% of healthy subjects have better and 50% have worse HRV values in the RSA measurement, was calculated [45,46].

### 2.5. Statistical Analysis

SPSS (IBM, Cary, NC, USA, Version Windows 20.0) was used for statistical analyses. A series of *t*-tests were performed for correlated measures comparing baseline and end of treatment measures for all parameters. Given the exploratory nature of this investigation, we did not adjust the *p*-value for significance for these tests. Effect size values were additionally calculated for each parameter and interpreted as recommended by Cohen 1988 to aid evaluation of the magnitude of any changes noted: 0.20–0.49—small; 0.50–0.79—moderate; and ≧0.8—large [47]. Effect sizes were reported to help interpret the clinical significance of the findings in this preliminary sample.

## 3. Results

### 3.1. Outcome: Pain Intensity

Average pain intensity decreased significantly as a function of treatment, with a large effect size, as did each of the four separate measures of pain intensity (as shown in Table 1).

### 3.2. Secondary Outcome: Pain Interference

Overall pain disability levels decreased a significant amount as a function of treatment, as did five of the seven individual components comprising this overall measure; with all revealing a large effect (see Table 2). Although changes with respect to “relations with other people” and “sleep” were not significant at the 0.05 level, the magnitude of change for each was large.

### 3.3. Process: 24-h HRV

Although rMSSD was the only measure to reveal a statistically significant change (with a large effect size), pNN50 (%), LF, HF, total frequency, and biological age nonetheless revealed medium effect scores, with Bpm and SDNN producing small effect sizes (see Table 3).

## 4. Discussion

The aim of this pilot study was to provide an initial assessment of the effectiveness of the IFT method for ameliorating pain and disability, as well as to determine concomitant changes in HRV activity. Our primary findings revealed IFT, a combination of MTPR and PBT, to be an effective treatment for chronic lumbar back pain, yielding statistically significant reductions in nearly all pain parameters—pain intensity as well as measures with bearings on quality of life and disability—with large effect sizes for all separate indices even for the few values that did not reach the conventional level of significance.

Only a single HRV parameter—rMSSD—revealed a statistically significant change among the nine parameters monitored, which stands in contrast to the more robust findings of Storella et al. [48] and Zhang et al. [49], who used surgical and chiropractic interventions and reported increases in various HRV parameters in patients who experienced decreases in pain. The fact that small to medium effect sizes were obtained for seven of our HRV parameters leads us to speculate that the absence of conventional statistical effects might well be attributed to our small sample size (*n* = 9). It is important to point out; however, that researchers are increasingly questioning the seminal value of significance testing, and instead are stressing the importance of focusing on magnitude of effects (along with precision of effects, such as confidence intervals) [50]. As our number of treatment sessions was relatively small and were applied over a brief period, we suspect that a greater number of sessions over a more extended period of time might have led to greater change. A greater duration of HRV treatment could potentially increase outcome values for the participants, statistical significance, and effect sizes. Treatment effects often take time to fully manifest. In future studies, patients will be engaging for an increasingly extended period of time.

The improvement observed with respect to rMSSD is notable, as it is associated with rapid changes in the HRV. This measurement is used to provide an overall indicator of parasympathetic tone or activity due to their strong, mutual relationship [51]. Should our results hold, therapeutic effects obtained with the IFT method may be due in part to stimulating the parasympathetic nervous system and aid in balancing the ANS. This can occur not only through the HRV training. A study conducted using spinal manipulation and myofascial manipulation techniques used HRV as a measurement to view ANS shifts, yet it was the myofascial work that was employed [52]. It was found that myofascial work can impact the branches of the ANS, particularly depending on the site of the techniques. After the manipulation of the lumbar and cervical areas, there was more parasympathetic activity, while the thoracic region seemed to activate the sympathetic branch [52]. An improvement in the parasympathetic system has been shown to impact myofascial tension and, in turn, trigger points of pain and, especially, stress [53]. In future inquiries with the IFT method, it will be useful to have groups participating in HRV training and myofascial manipulation as well as a group only focused on myofascial techniques in order to observe the impacts that the body work has individually on the ANS.

There exists a variety of treatment options for chronic low back pain. For example, yoga and other exercise forms, spinal manipulation, rehabilitation, and cognitive behavioral therapy were found to improve the effects of chronic low back pain [6], but only with moderate efficacy, however. Spinal manipulation, or osteopathic manipulation, was tested against a sham treatment by Licciardone [18]. While there was a significant change in pain sensation and physical functionality compared to no treatment, the results were comparable to the sham manipulation group. This similarity led to the lack of clarity around whether the positive results were due to the treatment function or due to placebo effects. An additional study found a significant shift in pain and functional status for participants with chronic back pain; however, the results are not clinically significant or relevant [7]. Here, it is unclear as to whether the positive effects of spinal manipulation were due to the placebo effect as well. This knowledge can be applied to future studies for IFT; including a non-treatment comparison group would be beneficial to understanding the true effect of this interdisciplinary method.

Issues with anxiety are incredibly common and often have a comorbidity with various mental issues, which have substantial impacts on everyday living [54]. In a study conducted by Magnon et al. [55], slow, deep breathing was examined for its efficacy in improving perceived anxiety as well as objective vagal tone, observed through HRV measures. The breathing technique resembles the one used in this current study, except that the inhalation was 4 s and the exhalation was 6 s. After a single treatment of 5 min there were significant changes in both the subjective and objective measurements. While this differs from our non-significant HRV results, possibly because the measures were taken only immediately after the breath session, it does show that types of breath work to train the HRV can improve overall anxiety and mental health [55].

Anxiety and overall mental health can also be impacted by quality of sleep. Increasing sleep quality has shown to significantly shift mental health for the better [56]. In a study working with insomniacs, it was found that slow-paced breathing, very similar to the tactic used in this current study, increased HRV significantly. The autonomic nervous system dysfunction and decreased vagal activity found in subjects with insomnia is improved with this breathing technique along with sleep quality [57]. Anxiety and sleep have a bidirectional relationship, i.e., as one increases or decreases so may the other [58]. It is because of this correlation that the effect of HRV on anxiety or sleep individually is unclear. This change correlates with the positive impacts on pain conditions [59]. Those suffering from chronic pain conditions have, in many instances of practicing breathing techniques that target heart rate variability, experienced reduced pain along with a decrease in anxiety and increased functional ability [59,60].

As the IFT method includes two distinct components, it is not possible to determine which aspect most accounted for the improvements obtained. Deep breathing in combination with the sensation of relaxation is known to have positive effects on the modulation of the sympathetic arousal and on the perception of pain [61]. Another study investigating the relationship between heart rate and pain severity (including only males) showed that increased pain severity produced an increase in heart rate [62]. Studies that directly compare effects of MTPR and PBT would be helpful in distinguishing the independent contributions of the two different interventions more precisely.

We were not able to assess the degree of at-home practice of PBT for HRV modulation, and differential rates of adherence by participants may have impacted our findings. A more definite method of tracking required HRV training adherence will be implemented in future studies. As paced breathing training was also carried out under the control and watchful eyes of the therapists during treatment sessions, we can assert that paced breathing was applied correctly at least during actual sessions. It is thus important to study attention to adherence more carefully in the future. Participating in manual therapy and biofeedback interventions can increase participant expectations and perceived benefits, potentially creating a placebo effect. Future studies could implement randomized controlled trials with a placebo treatment group, where participants receive similar intervention without such specified therapeutic techniques or effective biofeedback training. In addition, including self-report measures of expectations could assist in quantifying the potential placebo impact.

## 5. Conclusions

This study employed the newly developed interdisciplinary fascia therapy method intending to improve chronic lower back pain. Five weeks of treatment significantly decreased pain sensation and interference with livelihood, activity, and life quality. There was significant change in only one HRV parameter among nine. Significant pain reduction was found in the subjective BPI self-report measures. The improved quality of life, albeit with limited HRV changes, is an encouraging result, given the prevalence of the issue. As this is a pilot study, more research should be conducted in the future with a larger sample size, more groups differentiated from each other in treatment, and a longer treatment time period.

## Figures and Tables

**Table 1:** Overall pain intensity (BPI scores).

Parameter	N	Values Prior to Treatment Mean ± std. Deviation	Values After Treatment Completion Mean ± std. Deviation	*p*-Values	Cohen’s d
Pain Intensity	9	2.92 ± 1.59	0.49 ± 0.73	<0.001	1.96
Average Pain during last 24 h	9	2.89 ± 1.52	0.5 ± 1.0	<0.001	1.86
Worst Pain during last 24 h	9	4.78 ± 2.05	1.11 ± 1.62	<0.001	1.99
Least Pain during last 24 h	9	1.56 ± 1.42	0.11 ± 0.33	0.012	1.40
Pain Right Now	9	2.44 ± 2.11	0.89 ± 1.00	0.038	1.29

**Table 2:** Pain disability and interference (BPI).

Parameter	N	Values Prior to Treatment Mean ± std. Deviation	Values After Treatment Completion Mean ± std. Deviation	*p*-Value	Cohen’s d
Pain Disability	9	2.38 ± 1.99	0.31 ± 0.49	0.012	1.43
General Activity	9	2.78 ± 2.11	0.89 ± 1.69	0.03	0.99
Mood	9	2.33 ± 2.50	0.0 ± 0.00	0.023	1.32
Walking Ability	9	3.0 ± 3.32	0.33 ± 0.71	0.025	1.11
Normal Work	9	3.0 ± 1.94	0.33 ± 0.71	0.001	1.83
Relations with other people	9	1.33 ± 2.18	0.0 ± 0.0	0.10	0.99
Sleep	9	1.78 ± 2.33	0.33 ± 0.71	0.07	0.87
Enjoyment of Life	9	2.44 ± 2.60	0.33 ± 0.71	0.05	1.11

**Table 3:** Twenty-four-hour HRV measures. Note: ^a^ small effect size; ^b^ medium effect size; ^c^ large effect size.

Parameter	N	Values Prior to Treatment Mean ± std. Deviation	Values After Treatment Completion Mean ± std. Deviation	*p*-Value	Cohen’s d
Bpm (Beats per Minute)	9	75.17 ± 8.61	71.86 ± 8.61	0.075	0.39 ^a^
SDNN (ms)	9	142.31 ± 34.22	136.61 ± 22.66	0.536	0.20 ^a^
rMSSD (ms)	9	23.53 ± 5.44	28.79 ± 6.85	0.045	0.85 ^c^
pNN50 (%)	9	4.51 ± 3.25	7.46 ± 4.95	0.098	0.71 ^b^
LF (Low Frequency) (ms^2^)	9	342.82 ± 153.46	423.76 ± 135.81	0.150	0.56 ^b^
HF (High Frequency) (ms^2^)	9	76.76 ± 37.37	104.39 ± 57.61	0.092	0.57 ^b^
Total Frequency	9	1470.30 ± 554.13	1740.74 ± 494.35	0.201	0.52 ^b^
LogLF/HF	9	0.65 ± 0.16	0.66 ± 0.19	0.984	0.01
Biological Age (years)	9	46.91 ± 4.68	41.97 ± 8.07	0.064	0.75 ^b^

## Data Availability

The original contributions presented in the study are included in the article, further inquiries can be directed to the corresponding authors.

## Abbreviations

ANS	Autonomic nervous system
BPT	Paced breathing therapy
CLBP	Chronic low back pain
HRV	Heart rate variability
IFT	Interdisciplinary fascia therapy
MTPR	Myofascial trigger-point release
RSA	Respiratory sinus arrhythmia
